# Three Hours Ambulatory Blood Pressure: A Surrogate for Daytime Ambulatory Blood Pressure Assessment in the Pakistani Population

**DOI:** 10.7759/cureus.17433

**Published:** 2021-08-25

**Authors:** Hunaina Shahab, Hamza S Khan, Mayera Tufail, Azmina Artani, Aysha Almas, Hamad A Shah, Aamir H Khan

**Affiliations:** 1 Department of Medicine, Aga Khan University Hospital, Karachi, PAK; 2 Department of Cardiology, National Institute of Cardiovascular Diseases, Karachi, PAK

**Keywords:** white-coat hypertension, post-clinic blood pressure, ambulatory blood pressure monitoring, clinic blood pressure, daytime blood pressure

## Abstract

Background

Office blood pressure (BP) measurement is affected by the white-coat phenomenon and shows a weaker correlation with the gold standard ambulatory blood pressure monitoring (ABPM). To overcome this limitation, 24-hour ABPM is recommended by the guidelines for the diagnosis of hypertension. However, 24-hour ABPM is expensive and cumbersome, which limits its use in low to middle-income countries like Pakistan. We aimed to assess if an abbreviated ABPM interval can be utilized to diagnose hypertension effectively in our population.

Methods

A cross-sectional study, involving 150 participants as part of the Post Clinic Ambulatory Blood Pressure (PC-ABP) study, was conducted in the cardiology clinics. Participants ≥18 years of age, who were either hypertensive or referred for assessment of hypertension, were included. Blood pressure (BP) readings were taken with an ambulatory BP monitor over a 24-hour period. After excluding the first hour called the ‘white-coat window,’ the mean of the first six systolic readings taken every half hour during the daytime was calculated and was called systolic three-hour ABPM. Pearson correlation coefficients were calculated and Bland-Altman plots were constructed to determine the correlation and limits of agreement between mean systolic three-hour ABPM and daytime-ABPM. Receiver operating characteristic (ROC) curve for systolic and diastolic three-hour daytime ABPM and area under the curve (AUC) were analyzed for the level of accuracy in predicting hypertension.

Results

Of the 150 participants, 49% were male, and 76% of all were hypertensive. The mean age of participants was 60.3 ± 11.9 years. The mean systolic three-hour ABPM was 135.0 ± 16 mmHg. The mean systolic daytime ABPM was 134.7 ± 15 mmHg. Pearson correlation coefficient between mean systolic three-hour ABPM and mean systolic daytime ABPM was 0.85 (p-value <0.001). The limits of agreement were 18 mmHg to −17 mmHg between the two readings on Bland-Altman plots and the area under the curve of the receiver operating characteristic (ROC) was 0.96, suggesting that three-hour systolic ABPM is a good predictor of hypertension.

Conclusion

Three-hour ABPM correlates well with 24-hour ABPM in the Pakistani population. We recommend considering the use of this abbreviated ABPM to screen hypertension where a full-length ABPM cannot be used. Further studies can be conducted on a larger sample size to determine the prognostic implications of this shortened ABPM.

## Introduction

Office blood pressure (BP) measurement is affected by the white-coat phenomenon and shows a weaker correlation with the gold standard ambulatory blood pressure monitoring (ABPM) [1]. While ABPM is recommended for the diagnosis of hypertension, using it on every patient may not be the most cost-effective approach as it is expensive [2] and has limited availability. This is especially true for a low to middle-income country like Pakistan where more than 65% of its population resides in rural areas [3]. Similarly, the use of home BP monitoring is limited by cost, lower literacy levels, the widespread use of non-validated BP devices, and lack of knowledge about the utility of this method amongst physicians [4]. Therefore, ABPM and home BP for diagnosing hypertension in this population need careful consideration. Prior studies show that it is possible to reduce the length of the ABPM [5-7]. We aimed to assess if a shortened ABPM interval can be used as an alternate strategy to diagnose hypertension in our population.

## Materials and methods

Study site, population, and sample size

This was a sub-study of the Post Clinic Ambulatory Blood Pressure study (PC-ABP), therefore the methodology including sample size calculation has been published previously [8]. In brief, it was a cross-sectional study that was conducted in the outpatient cardiology clinic at the Aga Khan University Hospital (AKUH), Pakistan, starting in 2015. Ethical approval was taken from the AKUH Ethical Review Committee (3461-Car-ERC-15). We included patients who were ≥18 years of age, and were either hypertensive (with clinic systolic blood pressure [SBP] ≥140 mmHg and/or diastolic blood pressure [DBP] of ≥90 mmHg [9]) or referred for the assessment of hypertension. We excluded pregnant females, those participants taking non-steroidal anti-inflammatory drugs, or those having diarrhea.

Ambulatory Blood Pressure Measurement

After the clinic measurements were taken by the consulting physician using an automated and validated BP device (OMRON HEM 7221-E, M6 Comfort, Omron Healthcare Europe, Hoofddorp, The Netherlands), utilizing a standardized protocol [8,9], a 24-hour ABPM monitor (Model: 90217A, Spacelabs Healthcare, Snoqualmie, Washington) was attached to every study participant. This 24-hour ABPM monitor was programmed to record BP and pulse readings every half hour during the daytime and every hour during the nighttime. Every participant was instructed to record their activity in a diary provided to them and was asked to carry on with their routine lifestyle. A standard technique of the ABPM application was utilized for every participant with the correct size of the bladder cuff used. The side effects of the ABPM including skin irritation, pain, or bruising due to the cuff as well as a disturbance in sleep were explained clearly to the participants [10]. The ABPM monitor was attached to study participants during the regular clinic hours between 9 am and 3 pm.

Participants returned to the clinic after their 24 hours of the ABPM was complete and the data were then analyzed. All participants were given cash compensation to cover their logistics and travel costs. Since the study was conducted on actual patients visiting the cardiology clinic, an earlier clinic follow-up was scheduled if a major discrepancy was found between clinic readings and ABPM readings.

Three-Hour Ambulatory Blood Pressure Measurements

The ABPM was considered valid when ≥85% of readings were recorded [11]. The first hour of the ABPM monitoring with two BP readings referred to as the ‘white-coat window’ was excluded from the analysis. The next two BP readings taken over one hour were known as systolic and diastolic one-hour ABPM. The next four BP readings taken over two hours were systolic and diastolic two-hour ABPM. The next six BP readings taken over three hours were called systolic and diastolic three-hour ABPM. The mean one-, two-, and three-hour ABPM was compared with in-clinic BP, 24-hour ABPM, daytime ABPM, and nighttime ABPM.

Statistical Analysis

Stata version 12 (StataCorp, College Station, Texas) was used for data analysis. For continuous variables, mean and standard deviations were calculated whereas, for categorical variables, frequency and percentages were calculated. Mean in-clinic BP, 24-hour ABPM, daytime ABPM, nighttime ABPM, and one-, two-, and three-hour ABPM were calculated. Pearson correlation coefficients were calculated to determine the correlation between the mean one-, two-, and three-hour ABPM (systolic and diastolic) with the three systolic and diastolic components of 24-hour ABPM (overall 24-hour ABPM, daytime ABPM, and nighttime ABPM). The abbreviated reading with the highest correlation with daytime systolic ABPM was used in the final analysis. The Bland-Altman plots were constructed to determine the limits of agreement between three-hour ABPM (systolic and diastolic) and the daytime ABPM (systolic and diastolic). We categorized daytime ABPM into hypertension (SBP/DBP: ≥135/85 mmHg) and non-hypertension (<134/84 mmHg) based on the European Society of Hypertension guideline daytime ABPM value for the diagnosis of hypertension [12]. We generated a receiver operating characteristic (ROC) curve for systolic and diastolic three-hour daytime ABPM and analyzed its area under the curve (AUC) for the level of accuracy in predicting hypertension.

## Results

Of the 162 patients who fulfilled our inclusion criteria, 150 agreed to participate in the study. The mean age of the participants was 60.3 ± 11.9 years. Hypertensive patients were 76% of the total participants and 49% were males. The baseline characteristics of the population are listed in Table 1.

**Table 1:** Baseline characteristics Table 1 shows a comparison between the baseline characteristics of hypertensive patients and those referred for the assessment of hypertension (n = 150).

Variables	Hypertensive patients (n = 114)	Referred for the assessment of hypertension (n = 36)	p-values
Mean age in years (±SD)	62 (±11.3)	55 (±12.5)	0.001
Male gender (n, %)	52 (46)	21 (58)	0.136
BMI (±SD)	31 (4.6)	26 (6.9)	0.07
Dyslipidemia (n, %)	49 (43)	8 (22)	0.027
History of coronary artery disease (n, %)	40 (35)	5 (14)	0.016
Type II diabetes mellitus (n, %)	28 (25)	2 (6)	0.015
Smokers (n, %)	9 (8)	3 (8)	1.0
History of stroke (n, %)	1 (0.8)	1 (2.7)	0.49
Grade 1 to 3 chronic kidney disease (n, %)	3 (3)	0	1.0
On any antihypertensive medication (n, %)	106 (93)	8 (22)	<0.001

The white-coat effect was found in 38% (n = 43) hypertensive participants and 25% (n = 36) of the participants who were referred for the assessment of hypertension had white-coat hypertension.

The mean ± standard deviation (±SD) in-clinic SBP, DBP, and pulse readings were 152.3 ± 21 mmHg, 90.9 ± 12 mmHg, and 74.1 ± 15/min, respectively. The mean (±SD) overall 24-hour ambulatory SBP, DBP, and pulse readings were 130.9 ± 13 mmHg, 74.8 ± 9 mmHg, and 70.7 ± 13/min, respectively. The mean (± SD) daytime ambulatory SBP, DBP, and pulse readings were 134.7 ± 15 mmHg, 78.7 ± 15 mmHg, and 72.6 ± 12/min, respectively. The mean (±SD) nighttime ambulatory SBP, DBP, and pulse readings were 121.9 ± 18 mmHg, 68.8 ± 9 mmHg, and 63.8 ± 10/min, respectively. The mean (±SD) one-hour ambulatory SBP, DBP, and pulse readings were 142.2 ± 18 mmHg, 81.6 ± 12 mmHg, and 75.0 ± 16/min, respectively. The mean (±SD) two-hour ambulatory SBP, DBP, and pulse readings were 139.0 ± 16 mmHg, 80.6 ± 11 mmHg, and 74.6 ± 14/min, respectively. The mean (±SD) three-hour ambulatory SBP, DBP, and pulse readings were 135.0 ± 16 mmHg, 78.3 ± 11 mmHg, and 74.6 ± 14/min, respectively.

Pearson correlation coefficients between one-, two- and three-hour ambulatory BP, the three components of 24-hour ABPM, and in-clinic BP are shown in Table 2.

**Table 2:** Pearson correlation coefficient values Pearson correlation coefficient values between one-, two- and three-hour ambulatory systolic and diastolic blood pressure (one-, two- and three-hour ambulatory SBP and DBP), the three components of 24-hour ambulatory systolic and diastolic blood pressure (overall 24-hour ambulatory BP, daytime ambulatory BP, and night-time ambulatory BP), and in-clinic systolic and diastolic blood pressure (in-clinic SBP and DBP). The p-value is considered significant at <0.05. SBP, systolic blood pressure; DBP, diastolic blood pressure; BP, blood pressure.

Pearson correlation coefficient	In-clinic SBP	Overall 24-hour ambulatory SBP	Daytime ambulatory SBP	Nighttime ambulatory SBP
One-hour ambulatory SBP	0.60 (p-value <0.001)	0.74 (p-value <0.001)	0.77 (p-value <0.001)	0.55 (p-value <0.001)
Two-hour ambulatory SBP	0.53 (p-value <0.001)	0.76 (p value <0.001)	0.80 (p value <0.001)	0.54 (p value <0.001)
Three-hour ambulatory SBP	0.46 (p-value <0.001)	0.82 (p-value <0.001)	0.85 (p-value <0.001)	0.60 (p-value <0.001)
Pearson correlation coefficient	In-clinic DBP	Overall 24-hour ambulatory DBP	Daytime ambulatory DBP	Nighttime ambulatory DBP
One-hour ambulatory DBP	0.50 (p-value <0.001)	0.79 (p-value <0.001)	0.80 (p-value <0.001)	0.58 (p-value <0.001)
Two-hour ambulatory DBP	0.50 (p-value <0.001)	0.84 (p-value <0.001)	0.85 (p-value <0.001)	0.59 (p-value <0.001)
Three-hour ambulatory DBP	0.46 (p-value <0.001)	0.85 (p-value <0.001)	0.85 (p-value <0.001)	0.61 (p-value <0.001)

**Figure 1:**
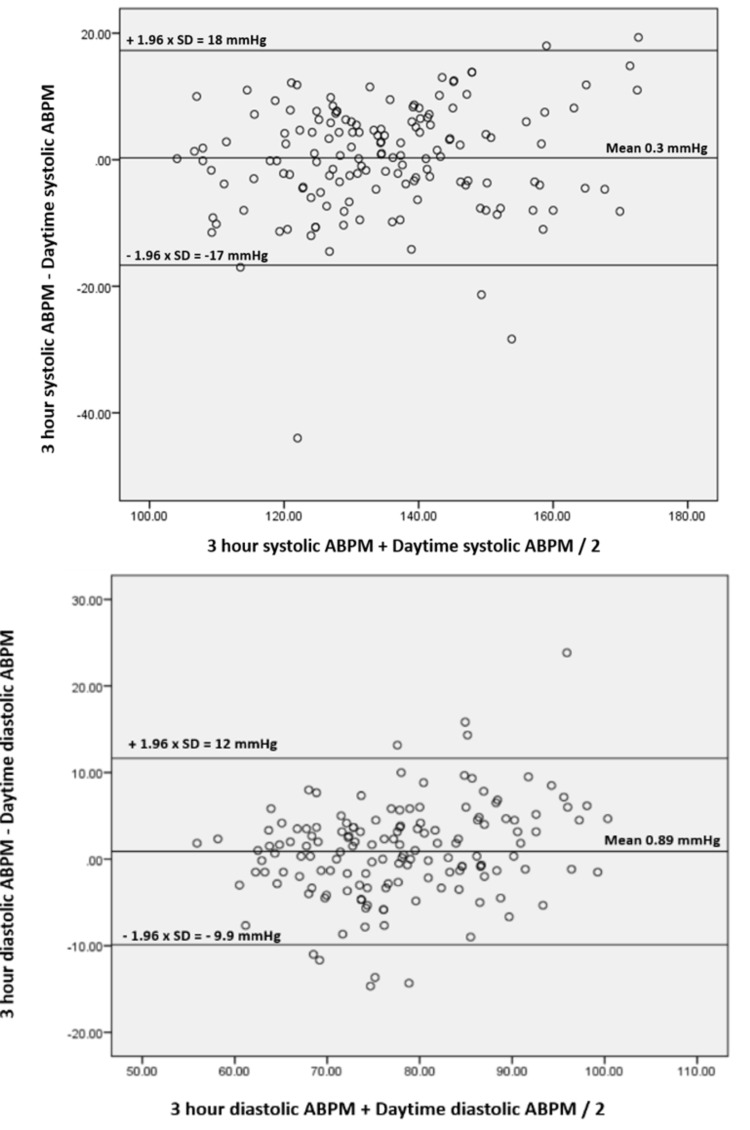
Bland-Altman plots Bland-Altman plots showing the degree of agreement between three-hour ambulatory SBP and daytime ambulatory SBP, and three-hour ambulatory DBP and daytime ambulatory DBP. SBP, systolic blood pressure; DBP, diastolic blood pressure; ABPM, ambulatory blood pressure monitoring; SD, standard deviation.

Based on the highest correlation shown between the three-hour ambulatory BP and the daytime ambulatory BP values, we chose three-hour ambulatory BP for the final analysis. Bland-Altman plots were constructed to show the limits of agreement between mean three-hour systolic and diastolic ABPM and daytime ABPM as shown in Figure 1.

**Figure 2:**
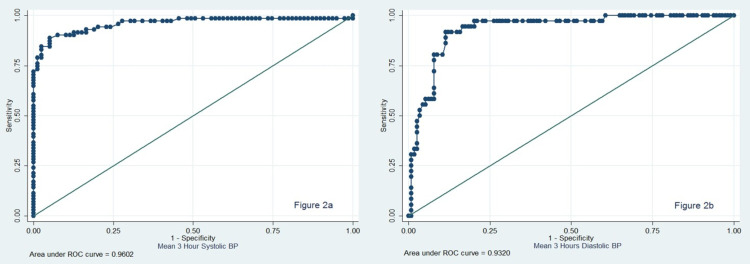
Receiver operating characteristic (ROC) curves a: ROC curve suggesting three-hour systolic ABPM to be a good predictor of daytime systolic blood pressure on a 24-hour ABPM measurement. Area under the curve is 0.96 (95% confidence interval = 0.92-0.99). b: ROC curve suggesting three-hour diastolic ABPM to be a good predictor of daytime diastolic blood pressure on a 24-hour ABPM measurement. Area under the curve is 0.96 (95% confidence interval = 0.92-0.99). ROC, receiver operating characteristic; BP, blood pressure; ABPM, ambulatory blood pressure monitoring.

Keeping daytime systolic and diastolic ABPM as the reference standard, the AUC of ROC was 0.96 (95% confidence interval = 0.92-0.99) for systolic three-hour ABPM and 0.93 (95% confidence interval = 0.89-0.98) for diastolic readings three-hour ABPM, suggesting three-hour ABPM to be a good predictor of daytime hypertension captured on a 24-hour ABPM, as shown in Figure 2.

## Discussion

In our population, three-hour ABPM has a strong correlation with daytime ambulatory BP, thereby making it a good predictor of daytime hypertension. Our results were similar to the study by Mas-Heredia et al. where they demonstrated that one-hour ABPM showed a good correlation with daytime ABPM [13]. Similarly, Weber et al. demonstrated a high correlation between an abbreviated two-hour BP interval with daytime and 24-hour ABPM [6]. Wong et al. also proved the utility of a shortened interval of 10-hour ABPM in the diagnosis of hypertension [14]. Amongst our patients, three-hour ABPM displayed the highest correlation with daytime and 24-hour ABPM in comparison to one-hour and two-hour intervals, therefore an interval of three-hour ABPM was considered superior.

Three-hour ABPM can offer several advantages in a resource constraint region. It can be useful in medical centers with limited resources where a single ABPM device can diagnose daytime hypertension according to the guideline-based recommendations in more than one patient per day, lowering the upfront cost of providing an accurate diagnosis. Furthermore, in patients who are unable to carry the ABPM for the entire day or find it uncomfortable, these patients can be advised three-hour ABPM to assess daytime BP levels. Physicians can quickly analyze the results of this shortened ABPM to make appropriate changes in medications within the same day. The three hours of ambulatory assessment can be spent outside the medical environment, thereby aiding the diagnosis of white-coat phenomenon and masked hypertension, as was possible with one-hour ABPM in Mas-Heredia et al.’s study [13].

Limitations

Three-hour ABPM may not be able to identify nocturnal dipping patterns, nocturnal hypertension, and the circadian variations in the BP, which may miss prognostically important information. However, the same information cannot be acquired with clinic BP or home BP readings as well. We have shown a good correlation between three-hour ABPM and daytime systolic ABPM. Thereby, we were able to determine a representative BP value within a shorter duration of monitoring to facilitate decision-making for the patient.

Despite the fact that a shorter interval of ABPM has shown reproducibility with daytime ABPM, it has not been studied for its prognostic value in predicting cardiovascular outcomes. Long-term follow-up studies with a larger sample size are needed to demonstrate the prognostic significance of a shorter interval ABPM and its utility in identifying patients who may have nocturnal hypertension.

Being a single-center study conducted in cardiology clinics, a generalization of our results may be limited. However, all the participants were Pakistani in origin so the results can be applicable to people of this region. Furthermore, our results were not stratified according to different antihypertensive medications, age, and gender of the participants, or the exact time of the day on which the ABPM was attached, which may show variable results.

## Conclusions

Three-hour ABPM shows a high correlation with daytime ABPM amongst the Pakistani population, making it a good predictor of hypertension. This shortened ABPM can be considered to screen and manage hypertension, especially where a full-length ABPM is undesirable due to cost or logistic constraints. Further studies can be conducted on a larger sample size to determine the prognostic implications of this abbreviated three-hour ABPM in hypertension management.
